# Comparisons Between Successful Versus Unsuccessful Switches From Benzodiazepines or Zolpidem (Z-Drug) to Controlled-Release Melatonin in Patients With Major Depressive Disorder and Insomnia

**DOI:** 10.3389/fpsyt.2020.00444

**Published:** 2020-05-19

**Authors:** Ji Hyun An, Mi Jin Park, Maurizio Fava, David Mischoulon, Hyewon Kim, Jihoon Jang, Jin Pyo Hong, Jun Sang Park, Hong Jin Jeon

**Affiliations:** ^1^Department of Psychiatry, Depression Center, Samsung Medical Center, Sungkyunkwan University School of Medicine, Seoul, South Korea; ^2^Depression Clinical and Research Program, Massachusetts General Hospital, Harvard Medical School, Boston, MA, United States; ^3^Department of Medicine, Ajou University School of Medicine, Suwon, South Korea; ^4^Department of Health Sciences & Technology, Samsung Advanced Institute for Health Sciences & Technology (SAIHST), Sungkyunkwan University, Seoul, South Korea; ^5^Department of Medical Device Management & Research, Samsung Advanced Institute for Health Sciences & Technology (SAIHST), Sungkyunkwan University, Seoul, South Korea; ^6^Department of Clinical Research Design & Evaluation, Samsung Advanced Institute for Health Sciences & Technology (SAIHST), Sungkyunkwan University, Seoul, South Korea

**Keywords:** major depressive disorder, insomnia, benzodiazepine, Z-drugs, zolpidem, controlled-release melatonin (Melatonin CR)

## Abstract

**Objectives:**

Chronic use of benzodiazepines and zolpidem (BDZ/Z-drugs) leads to dependency, cognitive impairment, and falls. Although controlled-release melatonin (Melatonin CR) may be an alternative, a switch in clinical settings has proven difficult. We investigated the factors associated with successful switch to Melatonin CR in patients with major depressive disorder (MDD) and insomnia.

**Methods:**

This retrospective observational study involved 170 patients diagnosed with MDD and insomnia, and aged older than 55 years, who were treated with BDZ/Z-drug for at least the past 90 days and never before exposed to Melatonin CR. All patients were treated with a standard antidepressant therapy and Melatonin CR for their insomnia. A successful switch was defined as three or more consecutive prescriptions of Melatonin CR separated by at least one-month interval, after discontinuation of the BDZ/Z-drug.

**Results:**

Seventy-nine patients (46.5%) who switched successfully showed no significant differences in sex, age, education, and occupational status compared with the unsuccessful group. The types of antidepressants used or BDZ/Z-drug treatment did not differ significantly between the two groups. Fewer somatic symptoms were observed in the successful group. In the multivariate analysis, the successful group showed significantly less somatic anxiety, shorter duration from first BDZ/Z-drug use to the first antidepressant use, and shorter total duration of BDZ/Z-drug therapy.

**Conclusions:**

A successful switch from BDZ/Z-drug to Melatonin CR was associated with less baseline somatic anxiety, earlier use of antidepressants, and shorter total duration of BDZ/Z-drug use, but was less strongly associated with the type of antidepressants in MDD patients with insomnia.

## Introduction

Insomnia is the key clinical characteristic of depressive disorders (1). Untreated sleep disturbance in depression can lead to significantly poorer clinical outcomes and a lower quality of life. Several epidemiological studies also revealed that insomnia increases the risk of type 2 diabetes mellitus, neurocognitive disorders, and obesity (2, 3).

Sleep disturbance is more prevalent in late life, estimated at approximately 30–40% (4, 5). The prevalence is more pronounced when depression accompanies sleep disturbance (6, 7). As a core symptom of depression, insomnia in later life has been associated with an increased risk of depressive episode relapse and suicidal ideation (8). The bidirectional relationship between depressive symptoms and insomnia is associated with complex comorbidity and is difficult to treat (6, 7). Treatment options for sleep disturbance are especially limited for the elderly because of their medical comorbidities as well as pharmacodynamic and pharmacokinetic differences. Also, insomnia in the elderly is easily regarded as part of the normal aging process.

Benzodiazepines (BDZ) or Z-drugs (nonbenzodiazepine drugs with benzodiazepine-like effects) are widely prescribed for insomnia, including elderly Major depressive disorder (MDD) patients. The rate of BDZ/Z-drug prescription has been reported up to 50.3% in the Korean population older than age 65 (9). These sedatives tend to be prescribed for insomnia as well as anxiety or physical pain before depressive symptoms are properly assessed. Even though the prescription is limited to 90 days by Korean law, the long-term use of BDZ/Z-drugs is a challenge (10, 11). The use of BDZ by the elderly is associated with several adverse effects such as higher accident rates, serious falls, cognitive impairment, ataxia, and even increased mortality (8, 12–14). Also, improper use of BDZ/Z-drugs could increase their abuse potential and dependence (15). Because of its immediate onset and anxiety relieving effect, however, prescription of the BDZ/Z-drug for insomnia is widespread in psychiatric and other medical practices, including primary care (16).

To address the inappropriate use of BDZ/Z-drugs, Melatonin Controlled - Release (CR) has been suggested as an alternative treatment for late-life insomnia, with a relatively safe profile and no rebound or withdrawal symptoms (17–19). Melatonin is a nocturnal hormone with circadian regulatory effects (20). Melatonin CR is a prolonged-release formulation of melatonin, designed to maintain the body concentration of melatonin during nocturnal periods (17).

Although many studies have demonstrated the effectiveness of Melatonin CR for primary insomnia, studies on its therapeutic effectiveness in insomnia prevalent in patients with MDD are limited. MDD patients can switch from BDZ/Z-drugs to Melatonin CR for insomnia safely without the risk of dependence and abuse.

Thus, the purpose of this study was to investigate the baseline predictors of successful BDZ/Z-drug discontinuation and change to Melatonin CR in MDD patients with insomnia, and to broaden the scope of clinical strategies for a successful switch in these patients. We hypothesized that fewer anxiety symptoms or less adjunctive antidepressant use predicts the successful discontinuation of BDZ in MDD patients with insomnia.

## Methods

### Study Subjects

This study is based on data from a retrospective, naturalistic observational single-center study conducted at the depression outpatient clinic of the Samsung Medical Center, Seoul, Korea. The study group comprised patients diagnosed with coexisting MDD and insomnia at their first visit and older than age 55 years since Melatonin CR is licensed for primary insomnia in that age group (17). From January 2009 to September 2019, this study recruited MDD patients with insomnia who had been prescribed a BDZ/Z-drug for at least 90 days, but with no previous Melatonin CR use. The 90-day period was required because the prescription for the BDZ/Z-drug was restricted to once per 90 days as the maximum in Korea. A minimum of 100 subjects were required for comparison between the two groups, assuming 80% power, 0.05 alpha, and an effect size of 0.55. A total target sample of 200 patients was selected and their electronic medical records were reviewed. Qualified psychiatrists assessed the patients according to the DSM-IV diagnostic criteria (21). Also, a trained psychologist independently performed neuropsychiatric testing to diagnose psychiatric disorders and the current mood state. Patients with the following conditions were excluded: comorbid bipolar disorder, schizophrenia, delusional disorder, eating disorder, substance use disorder, organic mental disorder, intellectual disability, neurological illness including epilepsy, serious medical illnesses such as terminal cancer, known allergic reaction to melatonin, and concomitant medications such as antibiotics including ciprofloxacin, steroids, non-steroidal anti-inflammatory drugs, which are associated with the risk of potential pharmacological interactions with melatonin. Thirty patients visited the outpatient clinic only once before initiating the Melatonin CR, and withdrew from this study before the evaluation to determine whether or not they should continue with the BDZ/Z-drug. Finally, a total of 170 MDD patients with insomnia were analyzed.

### BDZ/Z-Drug Discontinuation and Successful Switch to Melatonin CR

The decision to initiate Melatonin CR in the study patients was based on the agreement between the clinicians and individual patients in the clinical setting. Melatonin CR was initiated with a gradual reduction in BDZ dosage. All study patients were treated with 2 mg of Melatonin CR each for insomnia, per usual clinical practice. A successful switch to Melatonin CR was defined as three or more consecutive prescriptions of Melatonin CR each at least one month apart, after discontinuation of the BDZ/Z-drug (“successful group”). Otherwise, patients were assigned to the “failure group”. The prescription patterns of the BDZ/Z-drug including total duration, classification, and number of concomitant drugs used before initiation of Melatonin CR, were also measured.

All study patients received standard antidepressant treatment for the diagnosis of MDD. The following antidepressants were administered: escitalopram (10–20 mg/day), sertraline (50–100 mg/day), paroxetine (12.5–20 mg/day), mirtazapine (15–30 mg/day), venlafaxine (75–225 mg), desvenlafaxine (50–100 mg), bupropion (150 mg/day), or tianeptine (37.5 mg/day). The types and doses of the BDZ/Z-drug used before Melatonin CR initiation in study patients were: Lorazepam (0.5–1 mg/day), Clonazepam (0.5–1 mg/day), alprazolam (0.25–0.5 mg/day), etizolam (0.5–1 mg/day), diazepam (2–5 mg/day), zolpidem (5–10 mg/day), and zolpidem CR (12.5 mg/day).

Ethical approval was obtained from the Institutional Review Board of the Samsung Medical Center in Seoul, Korea (IRB number SMC-2017-11-059-004).

### Clinical Assessment of Psychiatric Symptoms and Laboratory Test

Sociodemographic information including age, sex, year of education, and occupational status were obtained at the first visit. Also, a baseline laboratory test was performed including complete blood count (CBC), lipids, chemistry and electrolyte profiles, inflammatory markers including c-reactive protein (CRP) and erythrocyte sedimentation rate (ESR), endocrine markers including follicle stimulating hormone (FSH), luteinizing hormone (LH), thyroid hormones, cortisol, and estradiol. The Hamilton depression rating scale (HAM-D) 17 (22), Beck depression inventory (BDI) (23), Hamilton anxiety rating scale (HAM-A) (24), and anxiety sensitivity index (ASI) (25) were initially used to measure the severity of depression and anxiety symptoms. The presence of insomnia was measured by the sum of the three items of HAM-D insomnia subscales (items 4–6), measuring initial, middle, and terminal insomnia, respectively. Each of the insomnia items was scored 0–2, resulting in a total score 0–6. To analyze the effect of anxiety on BDZ/Z-drug discontinuation, the mean HAM-A score for psychic anxiety (items 1–6, 14) and somatic anxiety (items 7–13), and the mean HAM-D score for anxiety/somatization factor (items 10–13, 15, 17) were also calculated (26, 27).

### Statistical Analysis

Sociodemographic variables and neuropsychiatric scores were calculated by Chi-squared analysis for categorical variables and Student's t-test for continuous variables. To evaluate the trend in continuation of Melatonin CR after discontinuation of the BDZ/Z-drug, the Spearman's correlation test of total days of Melatonin CR prescription on somatic anxiety score was performed. Next, we used the age- and gender-adjusted stepwise, multivariable regression model to predict the BDZ/Z-drug discontinuation. Depressive symptoms and anxiety scores were entered first in the analysis. Second, the total duration of BDZ/Z-drug use, the total time to the first prescription of antidepressants after the first use of the BDZ/Z-drug, and the frequency of concomitant use of BDZ were entered in the model. The above variables were calculated with a p-value < 0.05 based on univariable analysis. Variance inflation factors (VIF) of four or more as multi-collinear l and VIF with each variable in this study that did not exceed four were considered.

All statistical analyses were performed with IBM SPSS Statistics software Version 23.0 (IBM, Armonk, New York, USA). A statistical significance cutoff was set at an alpha level of 0.05.

## Results

### Sociodemographic and Clinical Characteristics of Study Patients

**Table 1** presents the sociodemographic and clinical features of all study subjects. Accordingly, 28.2% of the subjects were male and the mean age of the total cohort was 65.79 ± 8.15 (mean ± SD). Among the total of 170 patients, 79 (46.5%) successfully discontinued with the BDZ/Z-drug treatment and did not receive BDZ/Z-drug prescriptions for at least three months following Melatonin CR initiation. There were no differences in age, gender ratio, occupational status, and education years between successful and failure groups. The total duration of BDZ/Z-drug prescription until initiation of the Melatonin CR regimen was approximately twice longer in the failure group (752.82 days vs. 1,448.33 days), with statistical significance of *p* < 0.000. Also, the rate of concurrent use of BDZ/Z-drug therapy was significantly higher in the failure group (*p* = 0.002). The duration from the first prescription of the BDZ/Z-drug to the first use of antidepressant was significantly longer in the failure group (912.01 days) than in the successful group (593.28 days) (*p* = 0.012). Eight patients in the successful group and four patients in the failure group reported transient dizziness. There were no significant differences between the two groups in any of the baseline blood lab tests.

**Table 1:** Demographic and clinical characteristics.

Characteristics	Success group (n = 79)	Failure group (n = 91)	*p*-value
Male sex, N (%)	26 (32.9)	22 (24.2)	0.234
Age at time of registration, mean (SD), year	66.51 (7.97)	65.16 (8.29)	0.284
Occupational status, N (%)			0.058
None	26 (32.9)	16 (17.6)	
Occupation	19 (24.1)	23 (25.3)	
Homemakers	34 (43.0)	52 (57.1)	
Education, mean (SD), year	12.29 (3.86)	11.55 (4.23)	0.259
Total duration of BDZ/Z-drug (SD), day	752.82 (777.03)	1448.33 (1106.19)	0.000***
Time to the first use of antidepressant from starting the BDZ/Z-drug (SD), day	593.28 (745.24)	912.01 (885.87)	0.012*
Number of concomitant use of BDZ/Z-drug	1.62 (0.61)	1.92 (0.62)	0.002**

SD, standard deviation; BDZ/Z-drug, use of benzodiazepine or Z-drug.

*p < 0.05, **p < 0.01, ***p < 0.001.

### Increased Somatic Anxiety and Depressive Symptoms in the Failure Group

**Table 2** describes depression and anxiety symptoms in study patients. The failure group showed more severe symptoms of depression and anxiety than the successful group based on the HAM-D total score (16.99 vs. 14.51) and HAM-A score (19.69 vs. 14.65), respectively. Also, the failure group (26.05) showed higher self-reported depressive symptoms based on the BDI than the successful group (19.85). All these differences reached statistical significance (*p* < 0.05). In assessing the degree of insomnia, only early insomnia was significantly higher in the failure group (*p* = 0.042).

**Table 2:** Type of insomnia, depression and anxiety symptoms.

Characteristics	Success group (n = 79)	Failure group (n = 91)	*p*-value
**Type of insomnia, N (%)**			
1. Initial insomnia	51 (64.6)	74 (81.3)	0.042*
2. Middle insomnia	57 (72.2)	74 (81.3)	0.201
3. Terminal insomnia	43 (54.4)	61 (67.0)	0.115
**HAMD total score, mean (SD)**	14.51 (5.64)	16.99 (6.26)	0.007**
1. Insomnia, total	2.86 (1.64)	3.32 (1.74)	0.078
2. Anxiety/somatization factor, total	5.34 (2.11)	6.83 (2.33)	0.004**
**HAMA total score, mean (SD)**	14.65 (5.64)	19.69 (6.46)	0.000***
1. Psychic anxiety, total	9.37 (2.70)	10.17 (3.53)	0.111
2. Somatic anxiety, total	5.59 (3.00)	8.70 (3.30)	0.000***
**ASI score, mean (SD)**	18.31 (14.24)	21.12 (16.70)	0.329
**BDI score, mean (SD)**	19.89 (7.87)	26.05 (12.38)	0.002**

HAMD, Hamilton depression rating scale; HAMA, Hamilton anxiety rating scale; BDI, Beck depression inventory; ASI, anxiety sensitivity index; SD, standard deviation.

*p < 0.05, **p < 0.01, ***p < 0.001.

Interestingly, the HAM-D anxiety/somatization factors significantly increased in the failure group (*p* < 0.004) with higher somatic anxiety, gastrointestinal, and general somatic symptoms.

The increased somatic symptoms in the failure group were even clearer when somatic anxiety was measured by the HAM-A. Multiple somatic symptoms of anxiety including muscular, cardiovascular, respiratory, gastrointestinal, and autonomic were significantly higher in the failure group (*p* < 0.000). This trend was not observed in the sub-analysis of psychic anxiety.

### Characteristics of BDZ/Z-Drug and Antidepressant Use

**Table 3** describes the characteristics of each group depending on the BDZ/Z-drug and antidepressant therapy. Among various antidepressants, escitalopram was more likely to be prescribed in the failure group (49.5%) compared with the successful group (30.4%); however, no significant differences were found after Bonferroni corrections. The addition of mirtazapine, thought to be effective for BDZ/Z-drug discontinuation because of its higher sedating effect, did not show a significant difference between the two groups. Also, neither the prescription pattern of the BDZ/Z-drug based on the classification (anxiolytic including alprazolam and lorazepam vs. hypnotics including zolpidem and clonazepam vs. others) nor the lorazepam equivalent dose (0.62 mg for the discontinuation group vs 0.52 mg for the continuation group) showed statistical significance between the two groups.

**Table 3:** Characteristics of antidepressant or BDZ/Z-drug use.

Classification of adjunctive drugs	Success group (*n* = 79)	Failure group (*n* = 91)	*p*-value
**Use of antidepressant, N (%)**			
Mirtazapine	39 (49.4)	36 (39.6)	0.218
Duloxetine	4 (5.1)	11 (12.1)	0.174
Venlafaxine	3 (3.8)	1 (1.1)	0.339
Desvenlafaxine	9 (11.4)	4 (4.4)	0.146
Vortioxetine	8 (10.1)	4 (4.4)	0.229
Escitalopram	24 (30.4)	25 (49.5)	0.013
Fluoxetine	2 (2.5)	1 (1.1)	0.598
Paroxetine	8 (10.1)	8 (8.8)	0.798
**Antidepressant classification, N (%)**			0.107
NaSSA	38 (48.1)	36 (39.6)	0.281
SSRI	21 (26.6)	40 (44.0)	0.052
SNRI	9 (11.4)	8 (8.8)	0.616
others	11 (13.9)	7 (7.7)	0.218
**Use of BDZ/Z-drug, N (%)**			0.277
Alprazolam	18 (22.8)	20 (22.2)	0.900
Lorazepam	27 (34.2)	30 (33.0)	0.872
Zolpidem	16 (20.3)	9 (9.9)	0.081
Etizolam	6 (7.6)	7 (7.7)	0.981
Diazepam	1 (1.3)	1 (1.1)	0.920
Clonazepam	11 (13.9)	24 (26.4)	0.057
**BDZ/Z-drug classification, N (%)**			0.959
Anxiolytics	45 (57.0)	50 (54.9)	0.877
Hypnotics	27 (34.2)	33 (36.3)	0.872
Others	7 (8.9)	8 (8.8)	0.987

NaSSA, Noradrenergic and specific serotonergic antidepressant; SSRI, selective serotonin reuptake inhibitor; SNRI, serotonin-norepinephrine reuptake inhibitor.

*p < 0.0024(0.05/21). Bonferroni correction for multiple comparison

### Predictors of BDZ/Z-Drug Discontinuation in MDD Patients With Insomnia

**Figure 1:**
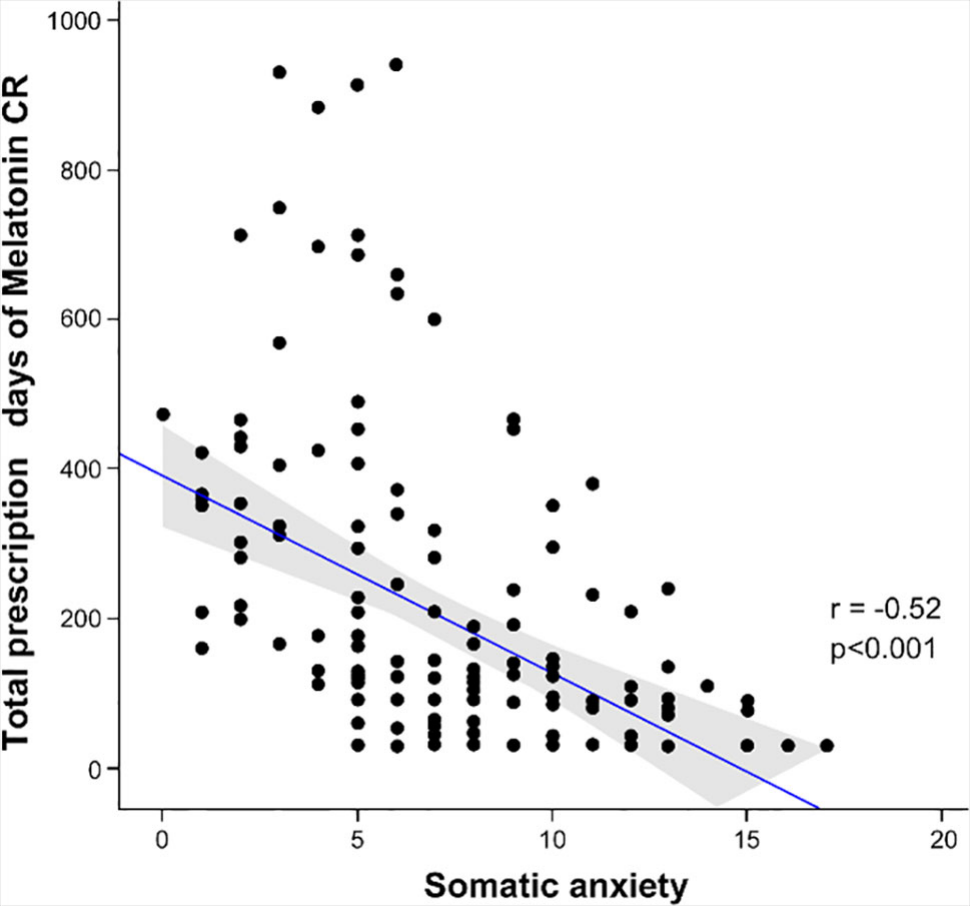
Scatterplot showing the correlation between total prescription days of melatonin CR and somatic anxiety score (Spearman correlation r = - 0.52, *p* < 0.001).

A significant negative correlation was found between the total prescription days of melatonin CR and somatic anxiety measured by the HAM-A with the r value of -0.52 and *p* < 0.001 (**Figure 1**). **Table 4** presents the factors associated with discontinuation of BDZ/Z-drug and successful switch to Melatonin CR in MDD patients with insomnia (univariate and multivariate analysis).Based on age and gender-adjusted stepwise multivariable logistic regression after entering statistically significant variables in the univariable analysis, Model 1 showed that a lower total HAM-A score [Adjusted OR (AOR) 0.86, 95% CI 0.80–0.93] and shorter total duration of BDZ/Z-drug use (AOR 0.99, 95% CI 0.99–1.00) predicted successful switch to Melatonin CR after BDZ/Z-drug discontinuation. Also in Model 2, fewer somatic symptoms measured by the HAM-A somatic anxiety items (AOR 0.74, 95% CI 0.64–0.83), shorter duration from first BDZ/Z-drug use to the first use of antidepressants (AOR 0.85, 95% CI 0.73–0.98) and less concomitant use of BDZ/Z-drug (AOR 0.51, 95% CI 0.26–0.97) were associated with successful switch to Melatonin CR. In the two multivariable models, the severity of somatic anxiety was more related to the BDZ/Z-drug discontinuation than the severity of depression.

**Table 4:** Factors associated with discontinuation of BDZ/Z-drug and successful switch to Melatonin CR in MDD patients with insomnia (univariate and multivariate analysis).

	Univariate analysis		Multivariate analysis			
			Model 1 (Age- and gender-adjusted)		Model 2 (Age- and gender-adjusted)	
	odds ratio (95%CI)	*p*-value	odds ratio (95%CI)	*p*-value	odds ratio (95%CI)	*p*-value
Age	1.01 (0.97-1.06)	0.49				
Gender, female	0.88 (0.42-1.85)	0.73				
Occupational status						
No	Ref	0.37				
Yes	0.61 (0.20-1.80)					
Homemakers	0.44 (0.18-1.09)					
Years of education	1.06 (0.97-1.15)		1.06 (0.96-1.18)	0.25	1.03 (0.93-1.15)	0.55
HAMD score, total	0.93 (0.88-0.99)	0.018*	0.98 (0.89-1.07)	0.65	–	
HAMD score, anxiety/somatization factor	0.78 (0.67-0.92)	0.003**	–		0.85 (0.69-1.05)	0.132
HAMA score, total	0.88 (0.82-0.95)	0.000***	0.86 (0.80-0.93)	0.000***	–	
HAMA score, somatic anxiety	0.73 (0.64-0.84)	0.000***	–		0.74 (0.64-0.83)	0.000***
Total duration of BDZ/Z-drug therapy(days)	0.73 (0.63-0.86)	0.000***	0.99 (0.99-1.00)	0.000***	0.99 (0.99-1.00)	0.000***
Time to the first use of antidepressant from initiation of BDZ/Z-drug (days)	0.83 (0.74-0.94)	0.003**	–		0.85 (0.73-0.98)	0.025*
Number of concomitant use of BDZ/Z-drug	0.46 (0.27-0.78)	0.004**	0.58 (0.30-1.13)	0.11	0.51 (0.26-0.97)	0.041*

HAMD, Hamilton depression rating scale; HAMA, Hamilton anxiety rating scale; CI, confidence interval; OR, odds ratio.

*p < 0.05, **p < 0.01, ***p < 0.001.

## Discussion

To our knowledge, this is the first study to investigate the independent clinical variables associated with discontinuation of BDZ/Z-drug and successful switch to Melatonin CR in MDD patients aged 55 years or older suffering from insomnia, in real-world clinical settings. This study showed that longer use of BDZ/Z-drugs and BDZ polypharmacy predicted an increased risk of continued BDZ/Z-drug use in MDD patients with insomnia, consistently with previous studies (28). Potential psychological and physiological dependence explain our findings, suggesting that the BDZ/Z-drug monotherapy should be used for a short period in such patients.

The continuation rate of the BDZ/Z-drug in insomnia patients varied according to the study design, estimates, and study populations from approximately 30–70% (11, 29). This discrepancy in the discontinuation/continuation rate may be attributed to a possible selection bias if the target sample was recruited from the general population or clinical setting, or because of the different study designs or methodologies. Our study population comprised clinical groups with depressive and anxious symptoms, with a higher proportion of the elderly especially vulnerable to BDZ dependence (15, 30), so the rate of BDZ/Z-drug continuation may have been higher than average. Also, the higher prevalence of anxiety symptoms after discontinuation of chronic BDZ/Z-drug therapy is attributed to rebound anxiety, withdrawal symptoms, or dependency.

We excluded other alternative psychotropic drugs for insomnia except antidepressants. As the antidepressants were initiated in MDD patients with insomnia earlier, the patients tended to discontinue the BDZ/Z-drug and easily switched to Melatonin CR. It is assumed that the BDZ/Z-drug is prescribed for an inappropriately lengthy period before depressive symptoms are assessed. This practice suggests that the adjunctive use of antidepressants in those patients is effective in improving insomnia as a depressive symptom as well as facilitating the discontinuation of the BDZ/Z-drug. Thus, the earlier evaluation of depressive symptoms and antidepressant initiation should be a priority among the elderly with MDD and insomnia.

We showed that MDD patients with insomnia who subsequently discontinued BDZ/Z-drug and successfully switched to Melatonin CR had significantly lower levels of somatic symptom anxiety compared with the failure group. Somatic symptom anxiety is strongly associated with MDD (26) and insomnia (31), and may be bidirectional (32). Thus, some elderly MDD patients with insomnia might benefit from appropriate treatment for somatic anxiety. Appropriate alternative drugs including antidepressants, non-BDZ anxiolytics or antipsychotics, and non-pharmacologic therapies could be used to relieve somatic anxiety related to insomnia (28, 29). A few studies even suggest that melatonin had potential to reduce somatic symptoms (33, 34). However, we were unable to demonstrate that melatonin reduced somatic symptoms in the study patients. Measuring the changes in clinical scores at the endpoint might facilitate the evaluation of the effectiveness of Melatonin CR for relieving somatic anxiety after discontinuation of BDZ/Z-drug.

Contrary to previous studies suggesting that hypnotics pose a higher risk of long-term BDZ/Z-drug use in the general population (10, 35), we found no differences in the risk of BDZ/Z-drug continuation according to their classifications as hypnotics vs. anxiolytics. Another group reported that the classification of the BDZ/Z-drug is not a predictor of treatment discontinuation (36). In patients with depression, anxiety symptom may be more prevalent than in the general population so that the use of anxiolytics might be higher without increasing the risk of chronic use of BDZ/Z-drug compared with hypnotics. However, the association between classification of BDZ/Z-drugs and the risk of their continuation is controversial.

There are limitations to this study. This study was restricted to a single-center clinic, mainly confined to older Korean patients, with a relatively small sample size. We were unable to fully confirm treatment adherence in terms of whether the medications were actually taken as opposed to simply having prescriptions filled. Therefore, the objective measures of sleep may have been accurate for the evaluation of improved sleep quality. Also, the three-month period of Melatonin CR maintenance may have been inadequate to evaluate possible relapses of insomnia symptoms later. Additional studies should be conducted over longer periods with larger clinical samples. Since only clinical variables related to BDZ/Z drug discontinuation were addressed, future studies focusing on the effectiveness of Melatonin CR for maintaining BDZ/Z-drug discontinuation during the withdrawal period should be performed, since the prolonged release of melatonin may potentiate the modulation of Gamma-aminobutyric acid (GAB_A_) receptor (37–39). Despite these limitations, our findings suggest a novel strategy of earlier management of somatic symptom anxiety and depressive symptoms, and that Melatonin CR was an alternative to BDZ/Z-drugs, and thus prevent the chronic use of BDZ/Z-drugs in elderly MDD patients.

## Conclusion

Melatonin CR is a safe substitute for BZD/Z-drugs in patients with primary insomnia as well as for patients older than age 55 with insomnia and accompanying MDD. Clinicians should be aware of the crucial role of earlier detection of depressive symptoms, control of somatic anxiety, and accurate identification of BDZ/Z-drug history in MDD patients with insomnia following discontinuation of inappropriate BDZ/Z-drug regimen.

## Data Availability Statement

The datasets generated and analyzed during the current study are not publicly available because we are preparing an additional article. However, they are available from the corresponding author upon reasonable request.

## Ethics Statement

This study was approved by the Institutional Review Board of the Samsung Medical Center in Seoul, Korea (IRB number SMC-2017-11-059-004).

## Author Contributions

JA participated in the study design, conception, data analysis, wrote the first manuscript drafting, and revised new drafts from co-authors. MP, HK, JJ, and JP participated in the study design and directed acquisition of the data. MF, DM, and JH conceptualized the study and revised the manuscript. HJ participated in whole study design and conception and manuscript drafting. All authors read and approved the final manuscript.

## Conflict of Interest

DM has received research support from Nordic Naturals. He has provided unpaid consulting for Pharmavite LLC and Gnosis USA, Inc. He has received honoraria for speaking from the Massachusetts General Hospital Psychiatry Academy, Blackmores, Harvard Blog, and Peer Point Medical Education Institute, LLC. He has received royalties from Lippincott Williams & Wilkins for published book “Natural Medications for Psychiatric Disorders: Considering the Alternatives.”

The remaining authors declare that the research was conducted in the absence of any commercial or financial relationships that could be construed as a potential conflict of interest.
